# Chronic Alcoholism

**Published:** 1888-01

**Authors:** 


### CHRONIC ALCOHOLISM.

The evil effects of what is called the abuse of distilled spirits are universally admitted. Their extent and fatality can scarcely be over-estimated. The number and mortality of the diseases, clearly attributable to this cause are well known to every practitioner of medicine. His experience brings almost daily before his eyes the terrible results of intemperance in their more obvious forms. Only those who have expressly inquired into the subject have an adequate idea of the social evils resulting from it, or of the degree in which they press upon every member of the community. Among those who have thus inquired, there is but one opinion as to the fact; that of all the causes which are at present conspiring to degrade the physical, moral and intellectual condition of the mass of the people, there is not one to be compared in potency with the *abuse* of *alcoholic liquors*.

Drunkenness, habitual or occasional, finds no defenders; but there is a wide margin between this and "dram drinking," which is regarded by many as absolutely safe or commendable, and consists in the proper use of ardent spirits as contra-distinguished from their excesses or abuses. Upon this point the friends and opponents of "moderate dram drinking" have joined issue, the latter asserting that the practice is more dangerous than open drunkenness, the fatality of them differing only in the time required to accomplish the ruin. The fierce flame enkindled by the insane appetite for strong drink, which quickly consumes the body, mind and conscience of the drunkard, is known in the list of diseases as Acute Alcoholism. By the name at the head of this article is designated another form of this same disease, not less destructive in its results.

Physiology and Pathology, as taught by their ablest expounders, declare that alcohol has no place in materia alamentaria; that its habitual presence in the animal economy is a disturbing element; that in whatever dose taken it is a poison *pro tanto*, and like lead, ergot, arsenic, opium and other like agents, its only "moderate use," its legitimate use is to counteract some diseased condition existing in the body.

The symptoms of the deleterious effects of this poison, now known as Chronic Alcoholism, are so insidious and present themselves in such a variety of forms that the cause of them long escaped observation. More careful study has formed and verified a fearful catalogue of diseases as the result of the liquor habit, involving the body, the mind and the morals.

The wide radiation of the effects of this poison finds its explanation in the fact that when introduced into the system it never is assimilated, it ministers to no function, it never becomes a component part of any tissue of the organism. It is introduced unchanged into the blood, and by the circulation is carried to the minutest cells of which the body is composed; its general stimulant effect upon living tissues is exhibited throughout, but is especially marked by its action upon the nervous system, not npon thec erebrum alone, but upon the nervous matter of the sympathetic and cerebral systems wherever found. For this neurine it has such remarkable affinity, that in the brain of animals poisoned by alcohol it is found in much larger proportion than in other tissues, or in the blood itself. This special relation between alcohol and nervous matter accounts for its peculiar power of exciting the nervous centers to augmented activity.

This fact is of fundamental importance as showing how directly and immediately the whole nutrition and vital activity of the nervous system must be affected by the presence of alcohol in the blood; the alcohol being thus specially drawn out of the circulating current by the nervous matter, and incorporated with its substance in such a manner as to change its physical as well as its chemical properties.

It is important to observe that this affinity is obviously such as will occasion the continued presence of alcohol in the blood which, even in very minute proportion, will modify the nutrition of the nervous substance more than any other tissue of the body: for the alcohol will *seek out* the nervous matter and will fasten itself upon it; just as other poisons whose results are more obvious to the senses, will localize themselves in particular organs or even in particular spots of the same organ.

These facts show an insidious toxic effect of alcohol even when taken in small quantities. Carried by the blood, it reaches, and alters or paralyzes the normal functions of the entire nervous system.

One of the first manifestations of its effect is the creation and continual growth of a morbid desire for an increase and repetition of the dose of the poison. By repeated indulgence the desire soon becomes so strong as to dominate the will and render the victim unable to resist it. With the will power thus enslaved by an appetite, the brain becomes changed in structure and function, the moral sense totters and falls, and most or all the crimes forbidden by the decalogue follow in regular gradation. The senses of feeling, tasting, hearing, smelling and seeing become blunted. Thickening of speech, trembling of the hands or limbs and unsteady gait show incipient paralysis of the motor nerves.

The sympathetic system of nerves evince corresponding disorder by the flushed and heated face or rubicund nose, alternatin g with cold, clammy skin and pallid cheek. The progressive paralysis of by intellectual qualities is shown first by occasional and later the permanent diminution of the mental grasp, the power of logical reasoning and power of moral control.

All this and much more are the regular and physical effects of the "social cup," "dram drinking," the "moderate use" of intoxicating liquors, and constitute the insidious and fearful *disease*—chronic alcoholism, which degrades a man to a hopeless physical, mental and moral wreck.

### ITEMS.

Dr. Robert Battey, of Rome, Ga., is off on his usual winter vacation, going this year to California.

PERSONAL.—Dr. A. B. Ashworth, of this city, has been appointed the Secretary of the Medical Association of Georgia, in place of the late Dr. James A. Gray.

CONGRATULATIONS.—Dr. Thomas B. Rider (A. M. C. 1886) of Morgan City, La., was married December 6th, 1887, to Miss. Irene B. Lynch, of La Place, Ala.

THE Anatomical Board of this State is working smoothly, and from the present outlook its members are led to believe that it will fully meet the expectation of its friends, and supply the needs of the teachers of the science of anatomy in this State.

ATLANTA SOCIETY OF MEDICINE.—At the last meeting the following officers were elected to serve during 1888: President, Dr. V. O. Hardon; Vice-President, Dr. W. S. Elkin; Secretary, Dr. L. H. Jones; Treasurer, Dr. E. Van Goitdsnoven; Reporters Drs. A. B. Ashworth, W. F. Westmoreland, Jr., H. P. Cooper.

THE January number of *Scribner's Magazine* opens the second year of its publication. The success of its first year is well known, and its second promises extremely well if we may judge by its prospectus of what is to come. The illustrations have steadily improved, and the publishers promise that during 1888 they will be better than ever. A prospectus will be sent to any one upon application to Charles Scribner's Sons.

A MOST novel, convenient and valuable business calendar for 1888 is the Columbia Bicycle Calendar and Stand, just issued by the Pope Manufacturing Co., of Boston, Mass. The calendar proper is in the form of a pad, containing 366 leaves, one for each day in the year, to be torn off daily. A portion of each leaf is left blank for memoranda, so arranged that the memorandum blank for any coming day can be turned to immediately at any time. The pad rests upon a portable stand, and when placed upon the desk or writing table the entire surface of the date leaf is brought directly and left constantly before the eye, furnishing date and memoranda impossible to be overlooked.

WE have the pleasure of announcing the marriage of Dr. H. P. Cooper and Miss Henrietta Tucker, daughter of the distinguished H. H. Tucker, D. D., ex-Chancellor of the University of Georgia, which occurred on Thursday, December 8th, at the residence of the bride's father in this city. Dr. Cooper is a surgeon of merit and a successful practitioner, and is the popular Professor of Chemistry in the Atlanta Medical College, the students of which institution welcomed his return from a short bridal excursion in a manner which testified their respect for him and their high appreciation of his ability.

It is rumored that other members of the faculty will soon go and do likewise. Our best wishes follow them all.

A NEW HOSPITAL FOR ATLANTA.—The Superior Court of

Fulton county has granted a charter to the Franklin Hospital Society, with Drs. Joseph P. Logan, W. F. Westmoreland, Messrs. S, M. Inman, John Keely, William A. Hemphill, G. L. Norman, L. D. Nelson, A. C. Bruce, Sidney Root, Charles A. Collier, J. R. Gray, David Mayer, Theodore Schumann, Revs. W. J. Gaines, and E. R. Carter, of Georgia, George W. Chapman, of Massachusetts, M. K. Jessup, H. M. Field and John D. Rockafellow, of New York, D. H. Burnham, of Chicago, and L. K. Fuller, of Vermont, as incorporators. We learn that a meeting has been held, and it was decided to commence and complete the work at an early day as possible. The hospital will be for the benefit of colored people only. There are very few places in this country where such an institution is more needed than Atlanta.

ROUND WORMS.—The following is recommended by De Grange:

R.—Santonin et hydrarg, chlor. mitis, aa gr. iv.

M.—Ft. cht. No. 1.

Sig. At bedtime. To be followed by a half ounce of castor oil early in the morning.

A PHYSICIAN in this city was last year prosecuted for homicide for administering to a child a dose of five grains each of calomel and santonin being only one grain each more than was in the above recipe.

After a full investigation and the introduction of numerous expert witnesses the prosecution failed.

MACON MEDICAL SOCIETY.—At a recent meeting of the Society, the following officers were elected to serve during 1888:

Dr. H. McHatton, president ; Dr. N. G. Gewinner, vice-president; Dr. E. G. Ferguson, secretary and treasurer; Dr. K. P. Moore, recorder; Dr. Howard Williams, reporter; Dr. C. H. Hall, lecturer.

A VOTE of thanks was given to Dr. E. G. Ferguson, retiring president. The society is in a most flourishing condition, and has a large and active membership.

CONTRIBUTIONS TO JOURNALS.—Any intelligent man who practices medicine for a decade or score of years acquires an experience of no small value to himself, and which can be made almost equally serviceable to his fellows if he only has the skill and the will to make it so. The large number of medical journals in the country do not leave him the excuse that he has no opportunity to communicate his knowledge to the world; and, in our opinion, he neglects a duty unless he seeks to avail himself of this opportunity.

Let us not be understood as urging all doctors to rush into print with heavy essays and ponderous articles; still less with lengthy and tedious reports of cases. But those facts and views, especially of diagnosis and treatment, to which they themselves attach great importance, can and ought to be common property. Short, pointed and practical articles, that are applicable and instructive for daily practice, are what are wanted. Results rather than methods, truths rather than suppositions, facts rather than theories, are asked from contributors.—*Mass. Medical Journal.*

THE disease of the throat, which for some time has afflicted the Crown Prince of Prussia, has excited the deepest interest throughout Christendom.

The high position and distinguished character of the sufferer have excited expressions of profound sympathy everywhere. Daily and weekly bulletins announcing the varying progress of the disease are read with great avidity.

His case is but a repetition of the history of that of another illustrious sufferer in our own country. The final fatality this last case has led the public to anticipate a like result in that of the suffering Prince. The feeling of surprise adds to the pleasure with which the last advices from San Remo have been received announcing a favorable change in his case, possibly justifying the hope of a final recovery.

Amid the sadness which pervaded this country during the illness of General Grant, the misunderstandings, the bickerings, the illy concealed rivalry or professional hostility among his medical attendants, afforded no little occasion for ridicule.

This element has not been wanting in the history of the treatment of the Crown Prince.

The differences of opinion between his distinguished English and German physicians and surgeons have attracted the notice of the profession in every civilized country, and have presented too fair a mark for ridicule to escape the shafts of the Gallic friends of Germany across the Rhine.

We accordingly see no end of witticisms at the expense of the rival specialists and laryngoscopists who direct the treatment of so important a case.

RABIES AMONG CHILDREN.—Alarmed at the increase of rabies among children, the Board of Health in Paris recently appointed a committee to inquire into the matter. The report by Dr. Oliver, recently published, attributes the frequency of hydrophobia among children to the fact that children, as a rule, are fond of teasing and playing with dogs, and that dogs suffering from rabies are peaceful during the incubation period, provided they are left alone, but their bite is none the less serious if irritated. To remedy this it has been decided by the authorities to issue a circular throughout both Paris and the provinces, enjoining parents, under various penalties enacted by the law, to keep a strict watch over their children to prevent them fondling or teasing dogs, and in every case of biting to report the fact immediately to the commissary of police, who will proceed at once to have the animal seized and, if necessary, killed. But would it not be easier and more effective, in France as in Germany, to carry out a universal muzzling order, effective over the whole country, and continuing for a considerable space of time?—*British Medical Journal.*

HYPNOTISM IN HYSTERO-EPILEPSY.—There is at present in the Bicêtre hospital an interesting case of hystero-epileptic attacks treated by hypnotism and apparently cured. The patient is a married woman of 23. The first hysterical attack occurred three months after her marriage; the second took place fifteen months later; from this time the attacks occurred two or three times daily, lasting from one and a half to two hours. They presented all the characterisics of hystero-epileptic attacks. It was necessary to tie the patient down and put on the strait waistcoat. She was subjected to energetic treatment, which failed to improve her condition. M. Paul Sollier finally resorted to hypnotism. He put the patient to sleep by pressure on the eyeballs, and induced somnambulism by friction of the vertex. He then suggested to her that she should have no more attacks for the next few days. The patient at first refused to accede to this request, but finally promised that she would have no attacks during the time named. This promise was literally carried out. The attacks then came on again, and M. Sollier again resorted to hypnotism with equal success. The patient returned to her home, but came to M. Sollier every fortnight, and subsequently every month, to be hypnotized. At the beginning of June, 1887, he suggested to her that she should never have any more attacks, and up to the present time these have disappeared. The patient occasionally complains of pain in the epigastrium, and shows a tendency to weep; she seemds to feel the need of hypnotism, but may be regarded as cured of her hysterical attacks. There is general insensibility to the prick of a needle, particularly on the left side, which is also insensible to the influence of cold or heat. Certain parts of the body are painful when touched—spots at the level of the left ovary, above and below the left breast, below the right breast, and at the level of the epigastrium. None of these points are characteristic of hypnotism or hysteria. The patient hears the tick of a watch held at 2 *centimetres* from her left ear, and at 10 *centimetres* from her right ear. With the right eye she can read a newspaper held at a distance of 5 or 6 *centimetres.* The sense of taste is diminished, especially on the right side of the tongue. The pharynx is completely insensitive. The sense of smell hardly exists on the left side. Muscular power is abolished on the left side, and patient appears altogether unconscious of her left arm. She is still troubled with hallucinations, which last five minutes at a time, during which she appears to see and speak to persons of her acquaintance. M. Sollier is of opinion that in cases of hystero-epilepsy, where the patient is hypnotizable and susceptible to suggestion, the attacks may be prevented by hypnotism.—*British Medical Journal.*

ANTISEPTIC PROPERTIES OF "LANOLIN" AS AN OINTMENT-BASIS.—Dr. Gottstein, in an article to be found in the *Berliner Klinische Wochenschrift* of November 28th, 1887, sums up the above subject by the statement that lanolin is absolutely inimical to the growth of micro-organisms, of whatever kind they may be. C. Fränkel had already stated that lanolin is free from germs, not only when prepared, but also in the raw condition of wool fat. It is well known that this substance does not undergo decomposition under the influence of the air, even when water or an alkaline solution is present, whereas all ordinary fats (glycerine fats) spontaneously decompose and become rancid from the separation of their appropriate fatty acids. Now, such decomposition of a body depends upon whether it is a fit material for the growth of bacteria or not, and it might, therefore, be asserted beforehand that lanolin is inimical to bacterial life. But Dr. Gottstein has submitted the question to direct experiment, and has found that no bacteria of any kind can be shown upon or in lanolin. Certain kinds of bacteria connected with putrefaction perish also in glycerine fats; and this is owing to two causes: first, because the fatty acids set free are hostile to bacteria; and secondly, because most germs found in the air are aërobiotic, that is, they require a fitting nutrition basis, the oxygen of air, for their development. The air, however, contains also anaërobiotic germs, and it can be shown by experiment that sterilized (ordinary) fat, after it has been exposed for a few days, and has undergone the usual changes (a yellowish appearance and rancid odor), contains anaërobiotic germs in its interior. This is one of the chief distinctions between lanolin and ordinary fats, for such germs are not found and cannot develop in lanolin. But a second series of experiments showed in a still more striking manner the difference in this respect between lanolin and ordinary fats. When bacteria develop in a certain nutriment, even though the latter undergo no visible alteration, they will penetrate into an underlying nutriment of fit character, and develop in it also. But if the layer into which they are first introduced be adverse to their growth, it will prevent their penetration into the adjacent layer of nutriment. Thin layers of different kinds of fats, including lanolin, were poured over the gelatine surface in various test tubes, respectively, and each tube, with its gelatine-covered fat, was thoroughly sterilized. After a few days anaërobiotic bacteria—or, rather, solid substances known to contain such bacteria, for example, garden mould and old cheese—were dropped upon the fat. The constant result was that bacteria rapidly developed in fat pork, and extended to the gelatine, while in the lanolin glasses the gelatine remained perfectly clear. The contrast was very striking. The results of the experiments, taken together, are as follows: 1. The bacteria which take part in the spontaneous decomposition of glycerine fats are presumably anaërobiotic. Of aërobiotic germs, several kinds (some of which are connected with ordinary putrefaction) perish upon a fatty nutriment basis, the rapidity with which they die being in direct proportion to the amount of fat present. 2. Exposed ordinary fat is found to contain anaërobiotic germs after a few days; lanolin similarly exposed contains no germs of any kind. 3. Glycerine fats can be penetrated by bacteria; lanolin, on the contrary, forms an impermeable layer. The above facts are of interest therapeutically and biologically. As regards biology, Liebriech has shown that cholesterine fats, which are chemically the same as lanolin, are a normal constituent of the epidermis in man and the lower animals, thus constituting the most powerful protection against infection from without. Furuncles may appear, it is true, and may be shown to contain pus-cocci; but no connection in point of time exists between this development in superficial or deeper layers of the skin. And furuncles are always a sign of deficient vitality of the skin; for example, in the stage of convalescence after typhoid, and in marasmic children. There must be a causal connection between these facts. Therapeutically speaking, lanolin has a great future before it. Its miscibility with water in any proportion, its ready absorbability by the skin, its freedom from any tendency to rancidity, as confirmed and explained by the above experiments, constitute it the vehicle *par excellence* for cutaneous medicaments. Its preservative properties ought also to find practical application in other ways.—*British Medical Journal.*

PERSONAL.—Dr. W. S. Kendrick, of this city, was married December 28th, 1887, to Miss Lula Groves, of Kartah, Ga.

DIAGNOSIS.—A friar once met some medical students, who asked him, "Do you know the difference between a friar and a jackass ? Give it up ?" "Yes, I give it up." "Well," said they, "one wears a cross on his breast and the other on his back." "Now," said the friar, "do you know the difference between a medical student and a jackass?" "No," said the students. "Neither do I," said the friar.

SUTURE OF THE URETHRA.—M. Lucas Championiere read before the *Societé de Chirugie*, seance of October 26th, the history of two cases of transverse ruptures of the urethra which had been treated by him by trimming off the lacerated portion and saturating the approximated ends. The results were excellent, and the distinguished reporter thinks that the urethra may frequently be sutured after wounds in which hitherto the suture has been neglected.

A CHINESE REMEDY FOR CROUP.—The formula of Tcheng-Ke-Tang is as follows: "Take from an old wall seven big spider webs, two of which at least should contain living spiders. Mash them up together (the webs and spiders), and add to the mass (or *mess*, we should say) about two scruples of alum dissolved in as little water as possible. Cook the whole over the fire until dry, and then increase the heat to incineration. The incinerated residue is powdered very fine and the powder is blown into the throat with a little bamboo cane. This is a sure cure." In short, the Chinese use burnt alum.—*St. Louis Medical and Surgical Journal.*

FOR CHAPPED HANDS.—A cream for chapped hands, which is far superior to many of the similar advertised products, is made as follows:

| | |
|---|---|
| Quince seed | ℥ij |
| Rose water | Oiv |
| Glycerine | Oij |
| Tr. benzoin | f℥ij |

Macerate the quince seed in the rose water for twenty-four hours. Strain, and add the glycerine and tr. benzoin.—*Drug. Bull.*

A MAN met his doctor on the street and complained of rheumatic pains, for which the doctor recommended him to take a pinch of "nitrate of potash" two or three times a day. Shortly afterwards he met the doctor again, and, in reply to a question regarding his health, remarked, "Oh, I'm getting well, doctor; but 'tain't your medicine. I tried that for some time, till a neighbor told me of something he took for his rheumatism—a very simple remedy. I tried it, and it does me a great deal of good." "What is it?" said the doctor. "Oh, it's simple; I'm afraid you'll laugh at me if I tell you." The doctor promised to control his risibles, and the patient, after much urging, informed him that it was "saltpetre." The doctor's smile was longer than the street. —*Chemist and Druggist.*

ARCHIVES OF PEDIATRICS.—The publishers are pleased to announce that in the issue of the January number, 1888, will begin a series of articles on the THERAPEUTICS OF INFANCY AND CHILDHOOD, by A. Jacobi, M. D., Clinical Professor of Diseases of Children in the College of Physicians and Surgeons, President of the New York Academy of Medicine, etc. The plan and scope of these articles are given in the following extract from Prof. Jacobi's letters to our editor: "I will prepare an essay on ten or twelve pages for every monthly issue of your journal. The subjects will be therapeutical. The first paper will probably contain general principles in their application to the disorders of early age. The following will treat of the therapeutics of the diseases of the newly born, of developmental and infectious diseases, those of the organs of circulation and respiration, genito-urinary organs, stomach and other abdominal viscera, muscles and bones, skin, nervous system, etc. Other subjects which will be treated of afterwards are certain classes of remedies, such as anæsthetics, narcotics, anti-febriles, purgatives, absorbents, roborants, and stimulants, etc. If there be time and room, the most interesting diseases, such as epilepsy, chorea, whooping-cough, and growths, may become the subjects of special papers.

IN an article on Intestinal Antiseptics, by D. N. Kinsman, M. D., appearing in the *Journal of the American Medical Association*, July 3rd, 1886, the author points out that the natural processes of fermentation and putrefaction going on in normal digestion, are so changed in dyspepsia and other forms of intestinal disease as to produce poisonous alkaloids, which are the cause of the symptoms developed in such disorders. The researches of Prof. Vaughn, of the University of Michigan, in which tyrotoxicon has been shown to be the cause of ice cream poisoning, which are still fresh in the minds of medical readers, have thrown still more light on the etiology of intestinal affections, and made apparent the importance of intestinal antisepsis as a method of treatment. To facilitate such treatment we learn that Parke, Davis & Co. have recently added to their list an Intestinal Antiseptic Pill, the formula of which is as follows: Mercury Protiodide, 1-8 gr.; Podophyllin, 1-16 gr.; Ext. Nux Vomica, 1-16 gr.; Ext. Henbane, 1-16 gr.

HOT WATER IRRIGATIONS IN GRAVE EPISTAXES.—Dr. Alvin, of Mont Dore, gives the history (in *La Loire Medicale*) of a case of epistaxes which had lasted forty-eight hours, resisting all methods usually resorted to, even complete plugging, until the patient was well-nigh spent. The doctor then bethought himself of hot water and by the aid of an English nasal irrigator, irrigated the nasal cavity with water heated to

65 or 70 degrees C. (150 to 160 degrees Fahr.) In three minutes the water came back clear and free from blood, showing that the flow was arrested. The irrigation was not painful, and as the upper lip was protected by a heavy moustache it was not burned by the water. The irrigation was repeated as a precautionary measure twice during the evening, and there was no return of hemorrhage. [The use of hot irrigations has long been resorted to in uterine hemorrhages and there is no reason why it should not be equally serviceable in epistaxis. There is no need of raising the temperature to the figure named, however, 115° to 120° Fahr. being quite sufficient.—EDS. ST. L., M. & S. JOURNAL.]

DIET IN SKIN DISEASE.—Dr James C. White, in an interesting paper on the above subject (*Journal of Cutaneous and Venereal Diseases*) states, among other things, that alchohol in excess may permanently enlarge the facial capillaries, produce dermatitis and furuncles, and other inflammatory troubles. Acid fruits produce in some persons acute eczema, the type being erythematous and papular. Strawberries produce urticaria. Apples sometimes produce an acneform eruption about the mouth. In children they produce the so-called "apple humor" at times. This consists of clustered vesicles or shallow impetiginous or ecthymatous lesions on the lower part of the face. Some nuts, especially the English walnut, produce an irritation of the lining membrane of the mouth in some. A herpetic eruption about the edges of the lips may also be caused. Lobsters, crabs, mussels, oysters, clams, etc., produce urticaria in a limited number of persons. In some again a particular meat has the same effect. These are important points to bear in mind, not only when prescribing a diet for a patient, but also in an examination, as they may often account for the skin trouble which is present.—*Medical and Surgical Journal.*

THE DRIFT CITYWARD.—The great, brilliant successes are, as a rule, in our cities. They attract notice. All men hear of the man who rolled up a fortune in a few years. Only few hear of the twenty that failed on the same lines. "What is hit is history; what is missed is mystery." One consequence is that the movement is from the country to the town. Young Thatcher is not going to plod along year after year on the farm when he might with less toil make his thousands in the city, as a politician or a man of business. "Why there is Baker—I'm just as smart as he is—and he is near the top of the wheel; they say he will soon be an alderman." So the tide is townward. Now, it is true that one may find the best people in the towns, for mind quickens mind; but you may also find the worst; and in this world evil works at a tremendous advantage. No better population for morals and trustworthiness is found in any Christian country than those who live by the tilling of the soil. We do not ignore the value of cities, but

"God made the country, and man made the town,"

and, without building on any forced exegesis of this passage, we cannot be blind to the fact that city life multiplies and complicates the problems with which Christian civilization has to deal. No five millions of country people in England present so much that is discouraging as you find among the same number crowded togethen in London.—*Rev. Dr. John Hall, in New Princeton Review for January.*

THE FOOD OF THE POOR.—Professor W. O. Atwater, in his article on the pecuniary economy of food in the January *Century*, writes as follows: "That the rich man becomes richer by saving, and the poor man poorer by wasting his money, is one of the commonest facts in daily experience. It is the poor man's money that is the most uneconomically spent in the market, and the poor man's food that is worst cooked and served at home. * * *

"I took occasion to make some inquiries myself among the Boston market-men, and one very intelligent butcher, in Boylston Market, said: 'Across the street over there is an establishment which employs a good many seamstresses. One of them comes to my place to buy meat, and very frequently gets tenderloin steak. I asked her one time why she did not take round or sirloin, which is a great deal cheaper, and she replied, very indignantly, 'Do you suppose because I don't come here in my carriage, I don't want just as good meat as rich folks have?' And when I tried to explain to her that the cheaper meat was just as nutritious, she would not believe me. Now Mr.—— and Mrs.——, who are among the wealthy and sensible people of this city, buy the cheaper cuts of meat of me. Mr.——very often comes and gets a soup bone, but I have got through trying to sell these economical meats to that woman and others of her class.'

"I am told that the people in the poorer parts of New York City buy the highest priced groceries, and that the meat-men say they can sell the coarser cuts of meat to the rich, but that people of moderate means refuse them. I hear the same thing from Washington and other cities. A friend of mine, a man of wealth, who, like his father before him, had long been noted as one of the most generous benefactors of the poor in the city where he lives, and with whom I happened to be talking about these matters, remarked: 'For my family I get the cheaper cuts of meat because they are cheaper. My children are satisfied with round steak and shoulder, even if they are not quite as tender and toothsome as sirloin. They are strong and healthy and understand that such food is good enough for their parents and is good enough for them.'

"I question whether his gardener or his coachman would be so entirely ready to accept such doctrine; and if the poor people to whom in times of stress his money is given without stint are like many others of their class, not a few of them would be ill content with some of the food-materials that appear regularly on his table."

THE TREATMENT OF URETHRITIS IN THE MALE.—In a paper read before the Medical Society of the County of Kings, Dr. H. W. Rand says that for painful urination in gonorrhœa small doses of the oil of sandalwood have given him better results than any other single remedy. He frequently combines ten minims of this oil with two or three grains of extract of hyoscyamus, and gives this quantity two hours after each meal. The effect is said to be more decided than when either remedy is given alone. As the inflammatory symptoms subside, the dose of the oil can be increased to twenty or thirty minims, and the hyoscyamus omitted. He also regards the oil of sandal-wood as the best internal remedy for the gonorrhœa itself. It is less apt to disagree with the patient than either copaiba or cubebs, and can be given earlier in the course of the disease. When the oil of sandal-wood is not tolerated, the tincture of *Cannabis Americana* can be given, and will be found a useful remedy. When pain on urinating is very severe, and is not relieved by the means already mentioned, injections of a four per cent. solution of cocaine are useful palliatives, if the act of injecting does not cause too much irritation.

Dr. Rand says that one of the best injections to begin with, which is soothing, and at the same time slightly astringent, is the following:

℞ Pulv. opii........................ʒi
Aquæ destill. bullientis..............f℥viii
Mix, filter, and add
Liq. plumbi subacetat.............fʒss

Sometimes a grain of sulphate of atropine may be profitably added to each ounce of this solution. As the inflammatory symptoms subside, the amount of solution of lead in the prescription may be increased to a fluid drachm or more, always bearing in mind that an injection should never be strong enough to produce pain, or even prolonged smarting. Not more than one fluid drachm should be injected early in the disease, but later on three or four drachms may be required to reach all the diseased surfaces. Treatment should be persisted in for at least two weeks after the discharge has entirely ceased—*Medical and Surgical Reporter.*

AN INTERESTING CASE OF POISONING BY TYROTOXICON.—Dr. V. C. Vaughan made a verbal report to the Michigan State Board of Health relative to the tyrotoxicon poisoning in Milan, Michigan, which presents some new features. He was called, September 25th, to see the family, two members of which were dying, and two others were seriously ill, while one other, a daughter, twenty years old, who had been away for some months, was not sick. The father had been sick about eight days, and the three others for a shorter time. The attending physician, suspecting that the sickness was due to tyrotoxicon, had warned the daughter when she returned not to drink milk. She complied, and was not sick. The mother and son died. He found the four patients breathing in a labored manner, the pupils of the eyes were dilated, and throbbing of the abdominal aorta was marked. The son was more or less delirious. All would pass urine involuntarily, but constipation was marked. Unfortunately, there was no thorough examination of the body after the first death; but upon the second death a thorough *post mortem* examination was held. Nothing abnormal was found, except that the large intestine was constricted by its circular fibres, which accounted for the constipation; the heart was arrested in systole; the death occurred from failure of the heart. The analysis of the stomach, by Professor Prescott, revealed no mineral poison. Dr. Vaughan's assistant, Mr. Novi, found enough tyrotoxicon in the contents of the stomach and intestines to sicken a cat.

Dr. Vaughan had confirmed the diagnosis of the attending physicians, who said it was sickness due to "cheese poison." It was not like arsenic poisoning, and, although it somewhat resembled poisoning by belladonna, yet there were points of dissimilarity enough to throw that out as a possible cause, especially as another poison was actually found in the stomach.

Dr. Vaughan soon made up his mind that the sickness was probably due to the bad and unwholesome condition of the house, which was fifty years old and nearly rotten. One floor was nearly rotted away, and was covered by a newer one. The house had settled a good deal; there was no cellar; the land in all directions sloped toward the house, so that the building was constantly on damp soil, as there was no artificial drainage. The sweepings and moppings for years had accumulated in the cracks of the floor so, that when the floor was taken up, a nauseating odor arose. The farmer sold cream to a creamery in the neighborhood, the proprietor of which had received the documents of the Michigan State Board of Health on cholera infantum and poisoning by cheese, milk, etc. He induced the farmer to keep his milk away from the house, and in a cool place, until the cream was collected and taken away. The milk consumed by the four members of the family was kept in a small closet or pantry in the house, where they frequently went and helped themselves to milk. They had been sick in the same way a number of times before this violent outbreak, which resulted in the death of two of their number. Dr. Vaughan made experiments as follows: He placed fresh milk in the pantry for a short time, and then found enough tyrotoxicon had developed in the milk to make a cat sick. He took some of the earth under the pantry floor and placed a small quantity of it in some fresh milk, soon after which tyrotoxicon was obtained from the milk, while none could be obtained from another sample of fresh milk which stood by the side of the milk in which the earth had been placed. This seems to demonstrate that the soil contained the germ of decomposition which produces the poison.—*Medical Record.*

THE DANGERS OF SANTONIN—From time to time I see reports on the action of santonin, which, in connection with my own experience, makes me doubtful of its harmlessness as an anthelmintic. I have been using it upwards of twenty years; many a time when strong symptoms of worms were present have administered santonin, and the signs of worms disappeared and the child became well, and yet no worm was seen. It is true I have nearly always exhibited the drug with the old, almost universal calomel. I have often met with cases of remittent and intermittent fevers in which the positive evidence of worms was present. In such cases have uniformly opened treatment with calomel and santonin in sugar, followed quickly with castor oil and spirits of turpentine. In such cases as the above, I often note a marked disposition to convulsions at once, and at last to gastro-enteric, or verminous fever. I have several times seen cases of obstruction of the bowel caused by the winding together of the dying worms caused by the use of santonin, and nervous and spasmodic trouble soon supervened. I have thought the use of santonin for the riddance of worms was more liable to such misfortune than any other vermifuge that I have used. Before I ever used santonin, and in the employment of other worm destroyers, I have often found a great variety of nervous symptoms and very threatening indications to arise as soon as the worms were disturbed or destroyed. Have not all practitioners, whether in the use of santonin or some other worm medicine, encountered some strange departures from the usual course of verminous disease? A malady that can cause night terror with all its phantasmagoric visions can also produce blindness, and has done so often when the stomach and brain have been long in morbid reciprocity by the presence of worms in the stomach. We may not be surprised at heart symptoms resembling the action of a powerful heart-depressant, or any other death-symptom which may have direct relation to the stomach or brain. My first use of santonin was in my own son, six years old. He had night terrors. I exhausted the catalogue of anthelmintics and tonics, mineral and vegetable. He got no better, but worse, until his frights at the sight of familiar friends at times threatened convulsions, and then temporary blindness was increasing in duration. I gave him santonin, per mouth and per rectum, morning and night, three grains each way. No worms appeared, and yet night-terrors stopped to return no more. A young man, twenty-one years old, clerk in a drug store, had night-terrors and consulted me on the subject. I ordered santonin and calomel each night for two nights. He laughed and said he was not wormy, and for a time refused to take the prescription; but at last he did take it, and his terrors ceased, though no worms appeared. I have had better success with santonin in the treatment for worms than any other vermifuge that I have used; have had as few strange departures from what is common to verminous diseases under its use as under any other worm medicine that I have used. I do hope its medicinal properties and therapeutics will soon be settled.—*Therapeuti. Gazette.*

FEMALE COMPLAINTS.—Did any one ever think how prolific a source of revenue to the medical profession are "female complaints," and how much forensic literature has been published, to enhance the value of certain operations, which have at first excited the admiration and afterwards the ridicule and disgust of the world? It is safe to say that more than half the revenue of the physicians of the world is derived from the treatment of females. And if we say that nine-tenths of the number thus treated have no need of that treatment, and that it works actual harm, we shall utter the truth. Not once in twenty times is the diagnosis correct; not once in fifty is the treatment successful (to the patient). Is it true that female complaints are increasing in such an enormous ratio to other forms of disease? And if so, what is the cause? Is it that as colleges and graduates in medicine and surgery (?) increase, there must be found new disorders of the sexual organs (to remedy the default of nature) to afford scope for the unbounded intelligence of the professors and room for the institution of a new professorship? The time must soon come when a healthy female will be a *rara avis in terris*, and the young man must provide a "hospital fund" before he takes a wife, certain to be obliged to call upon it in a few weeks or months. The ceremonials of gynecological attendance have also become something marvelous. The constant spraying, the ubiquity of disinfectants and antiseptics, the awe-inspiring instructions to the patient, the presence of an encyclopedic nurse, "with slow and solemn step," make the accouchement of a female something to be dreaded. Again, the diagnosis of any female complaint is not to be left to one individual. Nothing but a "consultation" of three or four long-visaged Esculapians will suffice, each one of whom afterwards thinks that *he* is the only one who fully understands that nothing was the matter, but that an operation of some kind must be had to satisfy the demands of profesional courtesy.—*Pacific Record of Medicine and Pharmacy.*

CASTRATION IN EPILEPSY.—In the *Berliner klin. Wochenschr.*, No. 3, 1887, S. Schramm reports two cases in which he performed castration for epilepsy. In the first case the ovaries were healthy, and the operation was done on account of the coincidence between the epileptic attacks and menstruation ; in the second case the ovaries were cystic, but the connection between the epileptic seizures and menstruation was not so positive. Immediately after the operation repeated convulsions occurred, but subsequently ceased entirely. In the first case there was no return of the disease within eighteen months ; in the second within a year after the operation.—*Medical Jaurnal.*

A METHOD OF ANTISEPSIS FOR THE URETHRA AND BLADDER. —At the meeting of the Academy of Medicine of Paris, October 29, 1886, M. Lavaux read a paper upon a method of antisepsis for the urethra and bladder, and its application to the treatment of strictures of the urethra. The following is a summary of his conclusions, as contained in the *Progrès Médical*, November 12, 1887: 1. Continuous *lavage* of the anterior part of the urethra and intra-vesical injections without a sound, constitute a simple and harmless method of accomplishing complete antisepsis of the urethra and bladder. 2. This method is applicable to the treatment of most strictures of the urethra. 3. Thanks to the complete antisepsis and the antiphlogistic action of hot injections into the bladder without a sound, complications due to rapid dilatation of the urethra are now very uncommon. 4. In the treatment of simple strictures which are easily dilated, rapid dilatation ought to be substituted for slow temporary dilatation, which is of but little value. 5. Intra-vesical injections made without a sound are sufficient to maintain the calibre of the dilated urethra. 6. Indications for the performance of internal urethrotomy become extremely restricted. 7. This method of treatment by securing antisepsis of the bladder and urethra ought to render divulsion and internal urethrotomy much less serious operations.—*Medical and Surgical Reporter.*

